# Biological dynamics and application prospects of Citri Reticulatae Pericarpium (CRP): a medicine and food perspective review

**DOI:** 10.3389/fphar.2026.1793560

**Published:** 2026-04-10

**Authors:** Angye Cai, Nan Diao, Xueqing Zhang, Junrong Zhang, Runrong Zhang, Chengfei Huang, Aygul Alim, Yuanbao Jin, Xin Zhao, Ke Feng, Wenzhong Hu, Sarengaowa

**Affiliations:** 1 College of Life Science, Zhuhai College of Science and Technology, Zhuhai, China; 2 Faculty of Medicine, Macau University of Science and Technology, Taipa, China

**Keywords:** chemical composition, Citri Reticulatae Pericarpium, different conditions, microbial activity, product application

## Abstract

Citri Reticulatae Pericarpium (CRP) is a plant widely used in medicine and food in China, offering extensive health-promoting benefits. Consequently, it plays a pivotal role in the healthcare sector and exhibits a variety of clinical effects, including anti-inflammatory, anticancer, cardiovascular protective, intestinal regulatory, and lipid-lowering activities. Its efficacy is attributed to a diverse range of chemical components. However, variations in geographical origin, storage duration, aging time, and harvesting period can lead to changes in these chemical constituents, thereby influencing the final flavor and commercial value of CRP. Currently, research in this field remains relatively fragmented. Therefore, this review summarizes the dynamic changes and mechanisms of action of the major bioactive metabolites in CRP based on these different factors. Concurrently, it analyzes the microbial activity and safety of the dominant strain, *Aspergillus* spp., and elaborates on the development and application prospects of CRP-derived products. The objective is to enhance the medicinal value of CRP in clinical therapy, explore its optimal flavor profile for food applications, and deepen its potential in innovative healthcare and daily chemical products. This work aims to promote the high-quality development of the CRP industry and propose further research directions for the innovative development and application of CRP-based products.

## 1 Introduction

**FIGURE 1:**
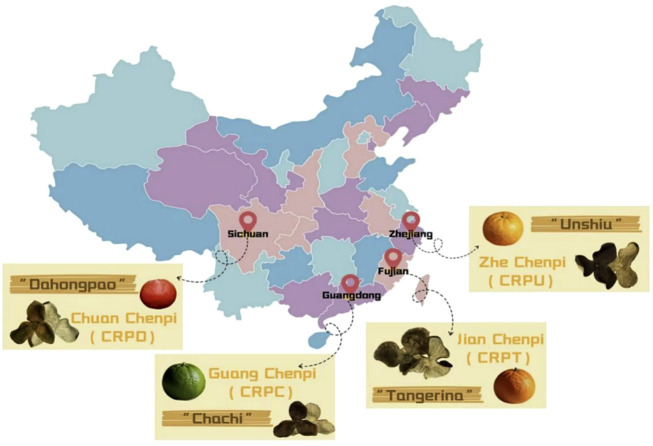
Regional distribution map of major CRP varieties in China. Abbreviation Notes: CRP refers to Citri Reticulatae Pericarpium.

Citri Reticulatae Pericarpium (CRP) is a common ingredient in food and traditional Chinese medicine, which is made from the mature peels of citrus (*Citrus Reticulata Blanco*) and its cultivated varieties by drying and processing (Wang et al., 2018). The geographical origin of CRP is widely distributed, featuring provinces, including Guangdong, Sichuan, Zhejiang, Fujian, and others (Figure 1) (Yu et al., 2018). Its skin is often peeled into several lobes, the base is connected, and the thickness is uneven (about 1–4 mm). The outer surface is orange-red or reddish brown, and the inner surface is yellowish white. The texture is thick, hard and crisp, and leathery (Yu et al., 2018). CRP is known as a high-quality traditional Chinese medicine, and its main effects are to regulate qi, strengthen the spleen, dry and damp, and dissolve phlegm. It has a long history of application, and it has been recorded in the *Shennong Bencao Jing* (also known as *Shennong’s Herbal Classic*) as early as the Qin and Han Dynasties (Zhang et al., 2024). However, because of the bitter taste of the peel, it is mostly used in the formulation of traditional Chinese medicines and not as a food.

The cultivars of CRP mainly include Guang Chenpi (*Citrus reticulata* pericarpium “Chachi”, CRPC), Chuan Chenpi (*Citrus reticulata* pericarpium “Dahongpao”, CRPD), Zhe Chenpi (*Citrus reticulata* pericarpium “Unshiu”, CRPU), and Jian Chenpi (*Citrus reticulata* pericarpium “Tangerina”, CRPT) (Figure 1) (Jiang et al., 2019). Among them, CRPC is the best CRP because of its excellent quality, while “Chachi” (also known as Xinhui CRP), grown in Xinhui City, Guangdong Province, represents the highest quality CRP. The shape of the CRP is neat but slightly sunken, with clear columnar markings. It is often separated into three connected petals, and is about 2.7–3.3 mm thick, with a large point oil chamber and a soft texture (Yu et al., 2018). Notable differences in appearance, oil cavity characteristics, and texture are observed among CRP varieties from different geographical origins (Table 1). Traditional CRP drugs are used to relieve inflammation, have antioxidant, anti-cancer, and antibacterial activities, and have the potential to promote health (Bian et al., 2022). Due to its unique aroma and taste, tangerine peel is often used as an ingredient in the food (Peng et al., 2019) and cosmetics industries, indicating its potential applications and benefits in different fields and thus highlighting the value of the citrus industry in health and life (Lin et al., 2003; Casquete et al., 2015).

**TABLE 1:** Characteristics of the main CRP varieties.

Producing areas	Varieties	Surface					Sensory characteristics
		Shape	Color	Oil cavity	Thickness	Texture	
Guangdong	Chachi	Three distinct valves, naturally curled margins, and closely attached pedicels	Brown-red to dark brown	Dense, uniform, and honeycomb-like	Thin (about 0.1–0.2 cm)	Soft	Rich and sweet, with a dried-fruit aroma; non-astringent even after prolonged cooking
Sichuan	Dahongpao	Obovate shape; broad peel; evidently separated lobes	Orange-red to dark-red	Sparsely distributed; uneven surface	Moderate (about 0.2–0.3 cm)	Hard	Pronounced fruity and citrus aroma; slightly astringent after prolonged cooking
Zhejiang	Unshiu	Indistinct lobe separation; flat edges	Dark yellow to light brown	Fine, flat, and smooth surface	Extremely thin (about 0.05–0.1 cm)	Fragile	Fresh citrus aroma; distinctly sour; prone to astringency after prolonged cooking
Fujian	Tangerina	Narrow and long lobes; pointed tips; distinct radial lines at the base	Orange-red to brown-red	Small and dense, with clear depression	Thin (about 0.1–0.15 cm)	Dry and fragile	Strong fragrance; slightly sweet; clearly graininess and astringent feel

In recent years, reviews have primarily concentrated on the properties and mechanisms of CRP’s chemical components. However, research on their dynamic changes and the resulting product efficacy remains insufficient. To clarify the research direction, this review aims to investigate the effects of factors such as geographical origin, storage duration, aging time, and harvest period on the chemical variations in CRP. Furthermore, it summarizes the medicinal health benefits and food industry applications of CRP within the context of its homology as both medicine and food, ultimately providing theoretical support for further in-depth research, development, and utilization of CRP in related fields.

## 2 Changes in the chemical composition of CRP

**FIGURE 2:**
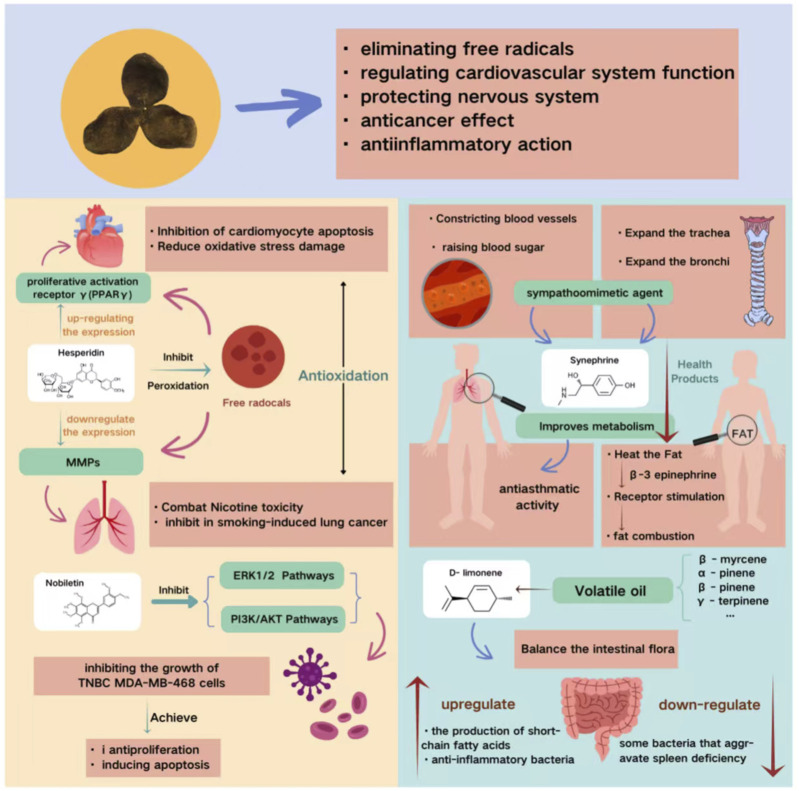
The mechanism of action of the main chemical components of CRP on disease treatment.

Through the ages, many scholars have studied the chemical composition of CRP in great depth and have found that it contains about 140 chemicals, including flavonoids, volatile oils, alkaloids, phenolic acids, terpenes, and tangerinel (Liu et al., 2013). CRP extract has been widely studied for its rich and unique phytochemical composition and has great commercial value (Mohammed et al., 2024); therefore, understanding its composition and how this affects its efficacy has certain potential to improve disease treatments and health (Figure 2).

### 2.1 Composition overview

#### 2.1.1 Flavonoids

Flavonoids are the primary bioactive metabolites of CRP and are mainly classified into three categories: flavonoid glycosides (including C-glycosides and O-glycosides), polymethoxyflavonoids (PMFs) (e.g., tangeretin and isosinensetin), and other flavonoids (e.g., hesperetin and naringenin) (Yu et al., 2018; Wang Q. et al., 2023). There is a strong consensus among domestic and international scholars regarding the therapeutic efficacy of these flavonoids (Yu et al., 2018; Stevens et al., 2019).

**FIGURE 3:**
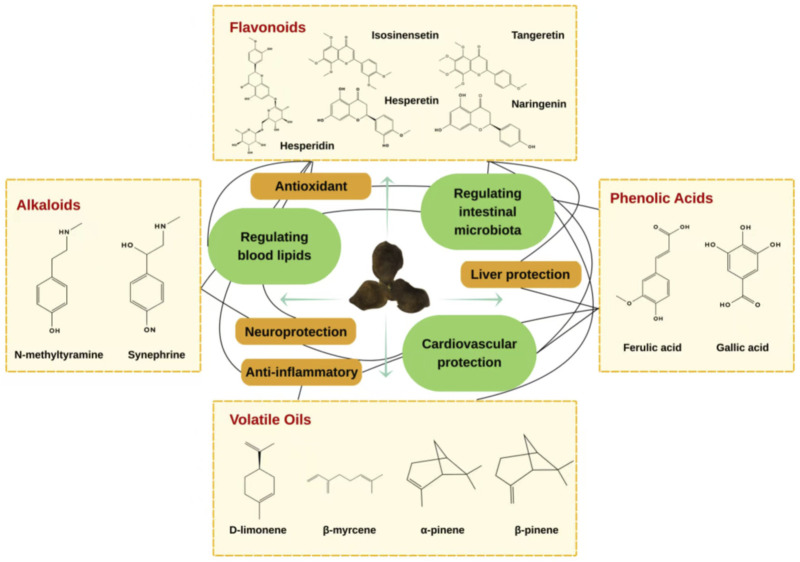
Chemical structure formula of the main active ingredients in CRP.

Hesperidin, a flavonoid O-glycoside and the predominant metabolites of flavonoid glycosides, exhibits a broad spectrum of pharmacological activities, including free radical scavenging, cardiovascular regulation, neuroprotection, and anticancer effects. The chemical structure of hesperidin has been systematically elucidated. Studies have shown that the hesperidin molecule consists of a flavanone backbone linked to a disaccharide unit (a disaccharide composed of glucose and rhamnose) via an O-glycosidic bond. Its molecular formula is C_28_H_34_O_15_, and the detailed structure is depicted in Figure 3 (Rosiak et al., 2023). The unique antioxidant properties of hesperidin are attributed to its structural features: the phenolic hydroxyl groups within the flavanone backbone can effectively scavenge free radicals via hydrogen donation or electron resonance mechanisms. Concurrently, the multiple hydroxyl groups in the disaccharide chain confer a significant metal-chelating capacity. By complexing transition metal ions (e.g., Fe^2+^), hesperidin inhibits the metal-ion-induced Fenton reaction, thereby indirectly reducing the generation of hydroxyl radicals (Abdelgawad et al., 2024). Its potent antioxidant activity effectively suppresses free radical-induced oxidative reactions and protects cellular structure and function by attenuating oxidative stress. Furthermore, studies have demonstrated that hesperidin can enhance antioxidant capacity by downregulating matrix metalloproteinases (MMPs), thereby antagonizing nicotine toxicity and inhibiting smoking-induced lung carcinogenesis (Balakrishnan and Menon, 2007). Fu H. L. et al. (2019) also reported that hesperidin exerts neuroprotective effects against depression by suppressing neuroinflammation.

Epidemiological evidence suggests a positive correlation between dietary flavonoid intake from CRP and reduced cardiovascular mortality (Mahmoud et al., 2019). This cardioprotective effect is partly attributed to the inhibition of cardiomyocyte apoptosis and the mitigation of oxidative stress damage via upregulation of peroxisome proliferator-activated receptor γ (PPARγ) expression (Agrawal et al., 2014). Similarly, nobiletin has been shown to exert antitumor effects by concurrently inhibiting the ERK1/2 and PI3K/Akt signaling pathways, thereby suppressing the proliferation and inducing apoptosis of MDA-MB-468 triple-negative breast cancer cells (Chen et al., 2014).

In summary, CRP represents a promising therapeutic agent, and its remarkable pharmacological effects are largely attributable to the synergistic actions of its constituent flavonoids (Zou et al., 2022).

#### 2.1.2 Alkaloids

Alkaloids are a class of nitrogen-containing basic organic metabolites widely distributed in nature, characterized by their unique low-molecular-weight structures (Idrees et al., 2021). The primary alkaloid identified in CRP are synephrine and N-methyltyramine (Zhang et al., 2024). The chemical structure of synephrine has been systematically elucidated. It is a phenolic alkaloid with the structure of 4-[1-hydroxy-2-(methylamino)ethyl]phenol and a molecular formula of C_9_H_13_NO_2_. Its molecular skeleton consists of a phenolic hydroxyl group and an ethyl side chain bearing a methylamino moiety (-CH(OH)-CH_2_-NHCH_3_) (Figure 3). This structure has been confirmed by nuclear magnetic resonance (NMR) and mass spectrometry (MS). Notably, synephrine contains a chiral center, and its stereochemical configuration critically influences its biological activity. This structure is analogous to that of adrenergic substances; however, compared to ephedrine, it lacks a hydroxyl group at the para position of the benzene ring and possesses only one methyl substituent on the side chain rather than two (Guimaraes et al., 2024). These structural differences determine its selective affinity for specific receptors. Synephrine is the most abundant alkaloid in CRP and has been demonstrated to possess a broad spectrum of physiological activities (Zou et al., 2022).

In the context of cardiovascular diseases, synephrine functions as a sympathomimetic agent, exhibiting vasoconstrictive and hyperglycemic effects, while also exerting significant dilatory effects on the trachea and bronchi (Yu et al., 2018). Several studies have further confirmed that synephrine extracted from CRP exhibits anti-asthmatic activity (Cao, 2012). Regarding endocrine system disorders, synephrine has been shown to enhance metabolic processes, increase caloric expenditure, accelerate fat decomposition and metabolism, and reduce lipid accumulation (Yu et al., 2018). As a naturally derived metabolites, synephrine is associated with minimal adverse effects. It promotes fat oxidation by increasing thermogenesis and stimulates β-3 adrenergic receptor-mediated fat breakdown, rendering it a common ingredient in weight management and related healthcare applications (Mattoli et al., 2005).

Studies have shown that N-methyltyramine is an N-methylated derivative of tyramine, with a molecular formula of C_9_H_13_NO and a structure corresponding to 4-[2-(methylamino)ethyl]phenol (Figure 3). This structure retains the phenolic hydroxyl group on the aromatic ring and the amine group on the side chain, conferring sympathomimetic activity that enables interaction with adrenergic receptors (Yokoo et al., 1999). Its N-methylated structure enables it to inhibit the α_2_-adrenergic receptor. This receptor antagonism leads to smooth muscle relaxation and increased intestinal peristalsis, contributing to the alleviation of functional gastrointestinal disorders (Koda et al., 1999). This finding was further corroborated by Fu M. Q. et al. (2019). Given that alkaloids constitute key bioactive metabolites underlying the pharmacological activities of CRP, their comprehensive investigation will substantially advance the development and utilization of CRP resources.

#### 2.1.3 Volatile oils

Volatile oils represent another prominent class of bioactive metabolites in CRP, comprising dozens to hundreds of volatile metabolites that are important metabolites of the CRP (Zheng et al., 2021). The total volatile oil content in CRP is estimated to range from 1.198% to 3.187%, with major constituents including D-limonene, β-myrcene, α-pinene, and β-pinene (Zhao and Lv, 2006). The chemical structures of these major constituents have been systematically characterized. They are all monoterpenes with the molecular formula C_10_H_16_, encompassing monocyclic, bicyclic, and acyclic olefin types (Figure 3). D-Limonene ((R)-limonene) is structurally characterized by a cyclohexene ring bearing an isopropenyl group (1-methylethenyl) at the C4 position, with the systematic name (4R)-1-methyl-4-(prop-1-en-2-yl)cyclohex-1-ene. This structural feature is responsible for its characteristic citrus aroma (do Santos et al., 2023). β-myrcene exhibits an acyclic structure, specifically 2-methyl-3-methylene-1,6-octadiene, containing three double bonds (two of which are terminal), classifying it as an acyclic monoterpene. In contrast, α-pinene and β-pinene possess a bicyclo[3.1.1] heptane skeleton (pinane skeleton). Their structural difference lies in the position of the double bond: α-Pinene has an endocyclic double bond between C2 and C3, whereas β-pinene features an exocyclic double bond between C2 and C10 (methylene group). This difference in double bond location results in distinct spatial configurations and, consequently, divergent physiological activities (Roviello et al., 2022). Among these, D-limonene is the most abundant, accounting for 75.1%–88.4% of the total volatile oil, followed by γ-terpinene (4.8%–13.5%) (Duan et al., 2016).

Volatile oils has been shown to improve gastrointestinal smooth muscle activity, accelerate gastric emptying, and enhance intestinal propulsion (Chen et al., 2021; Zhang et al., 2021). It also modulates intestinal flora by promoting the production of short-chain fatty acids and the growth of anti-inflammatory bacteria, while inhibiting bacteria that exacerbate spleen deficiency, thereby improving the intestinal environment (Zheng et al., 2020a). In addition, the volatile oils in CRP synergistically interact with other metabolites to exert a variety of pharmacological effects. For instance, they act in concert with flavonoids to significantly enhance the inhibition of lipid peroxidation, thrombosis, inflammatory responses, and endothelial dysfunction (Choi et al., 2015). In coordination with alkaloids, they contribute to the regulation of gastrointestinal function and exert expectorant effects, thereby enhancing the overall medicinal value of CRP. Beyond their contribution to the flavor and aroma of CRP, volatile oils possess substantial medicinal value, underscoring their diverse and important roles in both ecological systems and human health (Waris et al., 2022).

#### 2.1.4 Terpenes

Terpenes are widely distributed in plants, primarily as metabolites of essential oils, and represent the most common class of metabolites found in volatile oils, exhibiting similar bioactivities. As the main constituents of the volatile oil fraction of CRP (Yu et al., 2018), terpenes constitute an important material basis for its pharmacological activities. Li F. X. et al. (2021) successfully identified 33 terpene metabolites from CRP volatile oil using gas chromatography-mass spectrometry (GC-MS), among which D-limonene was the most abundant and serves as a typical representative of this class of metabolites.

Taking D-limonene as an example, its molecular structure consists of a cyclohexene ring with an attached isopropenyl side chain, conferring strong overall hydrophobicity. This structural feature enables it to readily penetrate and insert into the lipid bilayer of bacterial cell membranes. Upon incorporation, the cyclic skeleton and hydrophobic side chain of D-limonene disrupt the orderly arrangement of lipid molecules, increase membrane fluidity, and compromise membrane integrity. This leads to enhanced membrane permeability, resulting in the leakage of intracellular contents such as proteins and nucleic acids, and ultimately causing bacterial cell death (Lin et al., 2024). This mechanism also corroborates the structural basis for the broad-spectrum antibacterial activity of terpenes.

In addition to their antibacterial effects, studies have demonstrated that terpenes possess antioxidant, expectorant, and anti-inflammatory bioactivities, rendering them suitable as quality indicators for raw CRP materials (Zou et al., 2022). Based on these diverse biological activities and its distinctive aroma, CRP is utilized not only in traditional medicine but also finds extensive applications in the food and cosmetics industries, including as a food additive (Zhang et al., 2024), as well as in spices and essential oils (Zhang et al., 2020). To a certain extent, these diverse applications enhance the utilization rate and practical value of CRP in daily life.

#### 2.1.5 Phenolic acids

Phenolic acid metabolites are abundant in CRP, with 31 such metabolites having been identified to date (Wang Q. et al., 2023). Flavonoids, another major class of bioactive constituents, are characterized by a polyphenolic structure. In plants, phenolic acids and flavonoids can undergo interconversion and are metabolized through multiple shared pathways, which contributes to their similar biological activities. Phenolic acid metabolite confer numerous health benefits and medicinal properties upon tangerine peel, including antioxidant, anti-inflammatory, vasodilatory, antimutagenic, anticancer, and antibacterial activities (Rashmi and Negi, 2020).

Among these phenolic acids, ferulic acid exhibits particularly outstanding bioactivity. Ferulic acid is a phenolic acid metabolite with distinct structural features. Its chemical name is (E)-3-(4-hydroxy-3-methoxyphenyl) acrylic acid, and its molecular formula is C_10_H_10_O_4_. The molecular structure consists of a benzene ring core with a methoxy group (-OCH_3_) at the C3 position and a hydroxyl group (-OH) at the C4 position, linked via an acrylic side chain (-CH = CH-COOH) to a terminal carboxyl group (Figure 3) (Chen et al., 2019). This unique structure confers a dual antioxidant mechanism upon ferulic acid: on one hand, the phenolic hydroxyl group can directly scavenge free radicals through hydrogen donation, with the conjugated system facilitating stabilization of radical intermediates; on the other hand, the ortho-positioned phenolic hydroxyl group synergistically interacts with the carboxyl group, conferring metal-chelating capacity that enables complexation of transition metal ions (e.g., Fe^2+^, Cu^2+^), thereby inhibiting the Fenton reaction and indirectly reducing hydroxyl radical generation. Bian et al. (2022), Yang et al. (2022), Wang et al. (2016) have demonstrated the importance of this metabolite, confirming that CRP containing phenolic acids such as ferulic acid possesses potent antioxidant activity both *in vivo* and *in vitro*. Antioxidant capacity represents one of its core pharmacological effects, enabling direct scavenging of free radicals and effectively reducing oxidative cellular damage (Alshafei et al., 2023; El-Saadony et al., 2024).

Furthermore, the high content of phenolic metabolites significantly contributes to the anti-inflammatory properties of CRP, which are mediated by reducing pro-inflammatory cytokines and enhancing anti-inflammatory cytokine levels (Wu et al., 2018). At present, research on phenolic acid metabolites remains limited; further elucidation of their mechanisms of action would facilitate expanding the application prospects of CRP in pharmaceutical and food industries.

### 2.2 Metabolites changes

CRP, as a modern product bridging the gap between medicine and food, has high medicinal efficacy and edible value based on the richness of its chemical components. However, its chemical components vary significantly under different conditions, and factors such as different storage times, origins, and picking periods have an important impact on changes in its composition (Table 2) (Yu et al., 2018). Clarifying the trend in different conditions is conducive to providing a more accurate theoretical basis for CRP quality control and quality improvement.

**TABLE 2:** Summary of changes in major chemical components under different influencing factors.

Factors	Main chemical components	Molecular formula	Relative molecular mass	Variation trend and characteristics	References
Different storage time	Hesperidin	*C*_*28*_ *H*_*34*_ *O*_*15*_	610.56	Decrease (gradually converted to hesperetin)	Yang et al., 2022; Li et al., 2022
	Hesperetin	*C*_*16*_ *H*_*14*_ *O*_*6*_	302.28	Increase (product of hesperidin deglycosylation)	Yang et al., 2022; Li et al., 2022
	D-limonene	C_*10*_H_*16*_	136.23	Decrease (volatile loss and oxidation)	Gao et al. (2011)
	Polysaccharides	-	-	Decrease first, then remain stable	Wang Q. et al. (2023)
	Caffeic acid	*C*_*9*_ *H*_*8*_ *O*_*4*_	180.16	Increase	Choi et al. (2011)
	Gallic acid	*C*_*7*_ *H*_*6*_ *O*_*5*_	170.12	Increase	Choi et al. (2011)
	*p*-salicylic acid	*C*_*7*_ *H*_*6*_ *O*_*3*_	138.12	Increase	Choi et al. (2011)
	5-Hydroxyferulic acid	*C*_*10*_ *H*_*10*_ *O*_*5*_	210.18	Increase	Bian et al. (2022)
	3,4,5-Trimethoxycinnamic acid	C_*12*_H_*14*_O_*5*_	238.24	Increase	Bian et al. (2022)
	Ferulic acid	*C*_*10*_ *H*_*10*_ *O*_*4*_	194.18	Increase first, then remain stable	Choi et al. (2011)
	Benzoic acid	*C*_*7*_ *H*_*6*_ *O*_*2*_	122.12	Increase first, then remain stable	Choi et al. (2011)
	Cinnamic acid	*C*_*9*_ *H*_*8*_ *O*_*2*_	148.16	Decrease	Lim (2012)
	*p*-coumaric acid	*C*_*9*_ *H*_*8*_ *O*_*3*_	164.16	Increase first, then remain stable	Choi et al. (2011)
	Vanillic acid	*C*_*8*_ *H*_*8*_ *O*_*4*_	168.15	Increase first, then remain stable	Choi et al. (2011)
	Salicylic acid	C_*7*_H_*8*_O_*3*_	138.12	Increase	Choi et al. (2011)
Different aging time	D-limonene	C_*10*_H_*16*_	136.23	Decrease	Zheng et al. (2021)
	β-myrcene	C_*10*_H_*16*_	136.23	Decrease	Gao et al. (2011)
	α-pinene	*C*_*10*_ *H*_*16*_	136.23	Decrease	Duan et al. (2016)
	β-pinene	*C*_*10*_ *H*_*16*_	136.23	Decrease	Duan et al. (2016)
	γ-terpinene	*C*_*10*_ *H*_*16*_	136.23	Decrease	Duan et al. (2016)
	Hesperidin	*C*_*28*_ *H*_*34*_ *O*_*15*_	610.56	Decrease	Fu et al. (2017)
	Tangeretin	*C*_*20*_ *H*_*20*_ *O*_*7*_	372.37	Decrease	Fu et al. (2017)
	Nobiletin	*C*_*21*_ *H*_*22*_ *O*_*8*_	402.39	Decrease	Fu et al. (2017)
	Heptamethoxyflavone	*C*_*22*_ *H*_*24*_ *O*_*8*_	432.14	Decrease	Zeng et al. (2018)
Different producing areas	Tangeretin	*C*_*20*_ *H*_*20*_ *O*_*7*_	372.37	The highest content was found in CRPC, CRPC, CRPD > CRPU, CRPT	Jiang et al. (2019)
	Nobiletin	*C*_*21*_ *H*_*22*_ *O*_*8*_	402.39		Jiang et al. (2019)
	Hesperidin	*C*_*28*_ *H*_*34*_ *O*_*15*_	610.56	CRPC content above 2.0% (can be used to distinguish varieties)	Jiang et al. (2019)
	D-limonene	C_*10*_H_*16*_	136.23	CRPC>CRPD > CRPT>CRPU	Luo et al. (2018)
	γ-terpinene	*C*_*10*_ *H*_*16*_	136.23	The highest content was found in CRPC.	Zhou et al. (2009)
	Methyl 2-(methylamino)benzoate	*C*_*10*_ *H*_*13*_ *NO*_*2*_	179.22	Only the CRPC contains the same content	Zhou et al. (2009)
Different harvest period	Eleutheroside B	*C*_*17*_ *H*_*24*_ *O*_*9*_	372.37	The chemical components present in both mature and immature fruits can be analyzed to determine the optimal harvesting period for CRP.	Pontes et al. (2021)
	Icariside F2	*C*_*27*_ *H*_*30*_ *O*_*11*_	530.52		Pontes et al. (2021)
	Abietin	*C*_*16*_ *H*_*22*_ *O*_*8*_	342.34		Pontes et al. (2021)
	Sweroside	*C*_*16*_ *H*_*22*_ *O*_*9*_	358.34		Pontes et al. (2021)
	Verbenalin	*C*_*17*_ *H*_*24*_ *O*_*10*_	388.37		Pontes et al. (2021)
	Isosinensetin	*C*_*20*_ *H*_*20*_ *O*_*7*_	372.37		Pontes et al. (2021)
	Hesperidin	*C*_*28*_ *H*_*34*_ *O*_*15*_	610.56	Decrease	Zheng et al. (2021)
	Nobiletin	*C*_*21*_ *H*_*22*_ *O*_*8*_	402.39	Decreased significantly during immature stage, then remained stable during mature stage	Gao (2011)
	Tangeretin	*C*_*20*_ *H*_*20*_ *O*_*7*_	372.37		Gao (2011)
	3′,4′,5,7,8-Pentamethoxyflavone	*C*_*20*_ *H*_*20*_ *O*_*7*_	372.37		Gao (2011)
	4′,5,7,8-Tetramethoxyflavone	*C*_*19*_ *H*_*18*_ *O*_*6*_	342.34		Gao (2011)
	5-Hydroxy-6,7,8,3′,4′-pentamethoxyflavone	*C*_*20*_ *H*_*20*_ *O*_*8*_	388.37	Decreased significantly during immature stage, then increased significantly during mature stage, but remained lower than the immature stage	Gao (2011)
	Synephrine	C_*9*_H_*3*_NO_*2*_	167.21	Decreased	Zhang et al. (2020)
	Citrusinine-I	C_*16*_H_*15*_NO_*5*_	301.29	Decreased	Zhang et al. (2020)
	Germacrone	*C*_*15*_ *H*_*22*_ *O*	218.33	Decrease (The antioxidant activity was decreased.)	Wang and Liu (2014)

#### 2.2.1 Different storage times

The quality of CRP is widely recognized to improve with storage times (Lv et al., 2013). Over time, the peel loses its freshness, but its taste becomes richer and more complex, thereby enhancing its flavor and commercial value (Shi et al., 2024). Commercially available CRP is typically categorized by storage times ranging from 1, 3, and 5 years to over a decade (Li et al., 2024). Its value and market price increase proportionally with the number of storage years (Li et al., 2024). This phenomenon is primarily attributed to significant changes in the structure and content of chemical constituents within CRP during prolonged storage (Li A. L. et al., 2021). The main metabolites of CRP have been reviewed in 2.1.

The levels of flavonoids, the key bioactive constituents, tend to increase during the early stage of storage, followed by a gradual decrease until stabilizing within a certain range (Zheng et al., 2013). Specifically, Li et al. (2022), Yang et al. (2022) found that the content of hesperidin is inversely proportional to storage times, whereas the content of its aglycone, hesperetin, is directly proportional. It is worth noting that current research on the trends of PMFs, such as nobiletin and tangeretin, during storage remains inconclusive. Some studies (Fu et al., 2017; Wang et al., 2020; Yang et al., 2022) suggest that their contents increase in the early stages (within the first 3–4 years) and subsequently stabilize with minor fluctuations. However, other research (Li et al., 2022; Liang et al., 2022) indicates that the content of nobiletin and tangeretin does not significantly change with storage times. This discrepancy presents a valuable direction for future research into the relationship between CRP’s chemical composition and its quality.

Regarding volatile oils, Zhou et al. (2009) observed that their levels change slowly during the first 3 years of storage but fluctuate considerably over the subsequent 3 years, underscoring storage times as a key factor influencing CRP’s chemical profile. For instance, the concentration of D-limonene, the main metabolites of the essential oil, was reported to decrease from 454.7 g/L in the 3rd year to 378.7 g/L by the 17th year of storage (Gao et al., 2011). This reduction is significant, as high concentrations of D-limonene contribute to the unpleasant odor of fresh peel, and its gradual decline over time renders the aroma more acceptable—a finding consistent with earlier observations (Yi et al., 2006).

Changes in polysaccharide content follow a pattern similar to that of flavonoids, increasing during the initial storage phase before declining and eventually stabilizing (Wang Q. et al., 2023). Furthermore, a study by Gao et al. (2011) noted a decrease in polysaccharide content between 5–10 years of storage, after which it remained stable from 10 to 20 years, likely due to polysaccharide degradation over extended periods.

Phenolic acid metabolites play a pivotal role in enhancing CRP’s quality during long-term storage (Bian et al., 2022). The contents of various phenolic acids, including caffeic acid, gallic acid, salicylic acid, *p*-salicylic acid, 5-hydroxyferulic acid, and 3,4,5-trimethoxycinnamic acid, gradually increase with storage duration. Other metabolites, such as ferulic acid, benzoic acid, *p*-coumaric acid, vanillic acid, *p*-vanillic acid, and protocatechuic acid, reach relatively high levels after 5–7 years of storage and are maintained to a certain extent thereafter. Conversely, erucic acid, syringic acid, *m*-coumaric acid, and phenyllactic acid increase during the first 1–7 years but subsequently decline; nevertheless, their concentrations generally remain higher than those observed in the first year (Choi et al., 2011).

Notably, flavonoids are the primary active ingredients responsible for the antioxidant efficacy of CRP. The total flavonoid content in CRP from different storage years shows a significant positive correlation with ABTS, FRAP, and DPPH radical scavenging assays, confirming that increased flavonoid levels during storage enhance the antioxidant capacity of CRP (Liu et al., 2020). Several studies analyzing CRP samples from different storage years have concluded that longer storage durations are associated with superior antioxidant activity (Gao et al., 2011). At equivalent doses, the antioxidant effect of CRP stored for 6 years is significantly greater than that of samples stored for only 1 year, by more than threefold (Wang Q. et al., 2023). Consequently, CRP subjected to prolonged storage exhibits enhanced medicinal value and is typically preferred for applications aimed at reducing oxidative stress.

#### 2.2.2 Different aging times

A defining characteristic of CRP is its emphasis on “aging” (Chen). Throughout history, Chinese proverbs such as “the older, the more fragrant” have been widely circulated regarding CRP. The ancient traditional Chinese medicine classic, *Shennong’s Herbal Classic*, documented that prolonged aging enhances the quality of CRP. It is noteworthy that although both storage duration and aging time involve a temporal dimension, they are fundamentally distinct in terms of core objectives and environmental conditions, a distinction that must be clearly articulated. Storage typically refers to keeping materials under natural or uncontrolled conditions, with the primary goal of preventing spoilage and mildew to maintain basic preservability. In contrast, “aging” is a more proactive and refined process, defined as a time-accumulative progression under specific controlled environmental parameters. Wu et al. (2021) summarized the optimal parameters for CRP aging as a temperature of 25 °C, a relative air humidity of 65%, and a moisture content below 13%. (Wu et al., 2021). Its core objective is not merely preservation but the enhancement of CRP’s therapeutic efficacy and sensory quality through targeted chemical transformations, such as the gradual oxidative degradation of volatile oils and the accumulation of bound phenolic metabolites (Liu et al., 2024). This difference in objectives and conditions determines the irreplaceable role of the aging process in shaping the unique quality of CRP.

Investigations into the compositional changes during CRP aging have revealed that three-year-aged CRP contains abundant volatile oils (Duan et al., 2016), which are the primary active constituents responsible for its rich and mellow aroma. As noted above, the content of volatile oils gradually decreases with prolonged aging. Consequently, the aroma profile of CRP continuously evolves over time, gradually developing into a highly fragrant product that has become a popular ingredient in desserts (Shi et al., 2024). In fresh or conventionally stored CRP, the contents of flavonoids and bound phenolic metabolites are relatively low. However, under controlled conditions, the levels of these metabolites exhibit a gradual accumulation trend with extended aging time. The findings of Wang et al. corroborate this perspective: during the aging process of CRP stored in a cool, dry, and sealed environment, the contents of bound phenolic metabolites and flavonoids increased markedly, accompanied by a corresponding enhancement in antioxidant activity (Wang et al., 2016). Modern studies have further confirmed that aging time significantly influences the content of key bioactive metabolites in CRP, leading to variations in its diverse pharmacological activities (Zhang et al., 2024). Collectively, these findings underscore the critical role of aging duration in determining the medicinal and culinary value of CRP. Currently, research on the changes in other chemical constituents of CRP during aging remains relatively limited. Most existing studies have focused on the effects of general storage time alone, leaving significant research potential for future investigations into the dynamic changes specifically associated with the aging process itself, as well as comparative studies between storage times and aging times.

#### 2.2.3 Different production areas

The therapeutic efficacy of medicinal botanical drugs has been extensively investigated since ancient times (Zhang et al., 2024). According to the Tang dynasty medical text *Invaluable Prescriptions for Ready Reference* (*Bei Ji Qian Jin Yao Fang*), “the efficacy of medication depends on the geographical origin of the botanical drugs,” implying that the therapeutic effects of medicinal materials are determined by their production areas. This principle holds true for CRP, as the chemical composition of CRP cultivated in different regions varies due to distinct growth environments. These intrinsic chemical differences are externally manifested in the sensory characteristics of the peels (refer to Table 1) (Li et al., 2024).

Zhou et al. (2009) conducted an in-depth investigation of volatile oil profiles in CRP from different geographical origins and found that while their major compositions (including D-limonene and γ-terpinene) were qualitatively similar, the contents varied significantly. Notably, the γ-terpinene content in CRPC was significantly higher than in samples from other production areas. Moreover, CRPC contained methyl 2-(methylamino)benzoate, a metabolite not detected in CRP from other regions. Luo et al. (2018) reported that the volatile oil content was highest in CRPC (67.43 g/kg), followed by CRPD (6.74 g/kg), CRPT (4.50 g/kg), and CRPU (2.25 g/kg). Botanically, the oil cavities in CRP are classified as secretory cavities, which are specialized internal secretory structures. The primary function of secretory cavities is to store and secrete secondary metabolites such as volatile oils. These lipophilic substances accumulate within the cavity lumen and are typically surrounded by a layer of epithelial cells (Voo et al., 2012). This structural feature aligns remarkably with the description of CRPC in Table 1 as having “dense, uniform, honeycomb-like oil cavities,” indicating that greater oil cavity density corresponds to enhanced volatile oil storage capacity and higher volatile oil content. Volatile oils are the primary source of the distinctive aroma of CRP. The presence of methyl 2-(methylamino)benzoate, unique to CRPC, imparts a richer fruity aroma compared to CRP from other regions, and it does not easily develop astringency after prolonged cooking. This finding is consistent with the sensory characteristics of CRPC described in Table 1: “rich and sweet aroma, does not spoil after long cooking.”

Regarding flavonoid composition, the flavonoid content in CRPC is significantly higher than in other CRP varieties. According to the *Pharmacopoeia of the People’s Republic of China* (2020 edition), the hesperidin content in CRP must not be less than 3.5%. To better distinguish CRPC from other cultivated varieties, specific quality standards have been established using hesperidin, nobiletin, and tangeretin as reference markers: the hesperidin content should not be less than 2%, and the combined content of nobiletin and tangeretin should not be less than 0.42% (Zhang et al., 2024). Research findings indicate that the contents of tangeretin and nobiletin in CRPC and CRPD are higher than those in CRPU and CRPT. Further analysis using supercritical fluid chromatography coupled with a diode array detector revealed that, with the exception of CRPT, the tangeretin content in samples from the other three production areas ranged from 0.637 mg/g to 3.723 mg/g (Jiang et al., 2019; Liu et al., 2022). Wang et al. (2024) reached similar conclusions in their study. Among these, Ponkan (a variety of CRPD) exhibited the highest contents of nobiletin (5.26 mg/g), tangeretin (6.20 mg/g), and volatile oil (8.95%). Studies have shown that the browning index is significantly correlated with polyphenol content, suggesting that oxidative polymerization of flavonoids and phenolic metabolites may be one of the important mechanisms underlying color formation (Zhu et al., 2022). As shown in Table 1, CRPC and CRPD exhibit darker colors, while CRPU and CRPT appear lighter or brighter. This characteristic is closely associated with their higher accumulation of flavonoids, particularly nobiletin and tangeretin.

Due to these compositional differences, CRP produced in Guangdong Xinhui and Sichuan exhibits superior antioxidant properties compared to products from Fujian, Zhejiang, Hunan, and other provinces, with CRPC demonstrating the strongest antioxidant activity (Chen and Kitts, 2017). Consequently, CRPC is generally preferred due to its superior peel quality and chemical composition, ranking first among all varieties in terms of its medicinal and culinary value. However, citrus resources are widely distributed in China with numerous varieties. With the advancement and popularization of citrus breeding technology, coupled with the increasing maturity of CRP development and utilization, CRP from more production areas may possess unique potential warranting further investigation. Current research has primarily focused on CRPC, with relatively insufficient attention given to CRP varieties from other regions. Similarly, the intrinsic relationship between the sensory characteristics of CRP and its chemical composition represents a valuable research direction that has yet to be systematically and thoroughly explored.

#### 2.2.4 Different harvest periods

CRP harvested during different periods varies significantly in its chemical composition, content, and proportions, leading to differences in its medicinal properties (Zhang et al., 2020). CRP is typically harvested between September and December each year, and the harvest period is generally divided into three stages. The harvest from September to October is referred to as Qingpi (Qinggan), during which the fruit is unripe, with green and firm peel and a strong aroma. The harvest in November is known as Erhong pi (Erhong-gan), when the fruit is just ripe; the peel is predominantly turquoise with some yellow coloring, slightly cracked, and exhibits a strong aroma. The harvest from December to January is termed Dahong pi (Dahong-gan), during which the fruit is fully ripe, the peel is golden to orange-red, cracked, and emits a fragrant and mellow aroma.

Some researchers have used ethyl acetate and methanol as solvents to extract metabolites from the peel of mature fruits harvested between September and December and immature fruits harvested between July and August (Xiao et al., 2024). Their results indicated that the peel of mature fruit contains characteristic metabolites comprising nine metabolites, including six glycosides (eleutheroside B, icariside F2, abietin, sweroside, didymin, and verbenalin), two flavonoids (luteolin and isosinensetin), and one phenolic metabolite (4-(3-oxobuty)phenyl 6-O-[(2E)-3-(4-hydroxyphenyl) prop-2-enoyl]-2-O-(3,4,5-trihydroxybenzoyl)-beta-D-glucopyranoside). In contrast, the peel of immature fruit displayed characteristic metabolites consisting of 13 metabolites, including six flavonoids (7,8-dihydroxy-5,6-dimethoxy-2-phenyl-4H-chromen-4-one, isoosajin, isosinense, 4-oxo-3-phenyl-4H-chromen-7-yl 2-methylbenzoate, baicalein, and 2-(2,3-dihydrobenzo[b][1,4]dioxin-6-yl)-6-methylchroman-4-one), five glycosides (eleutheroside B, abietin, sweroside, icariside F2, and verbenalin), one alkaloid (eupalinilide D), and one terpenoid (germacrone) (Pontes et al., 2021). Moreover, antioxidant activity measured via the FRAP method revealed that the later the harvest time, the lower the antioxidant activity of terpene metabolites. This finding further confirmed that the content of terpene metabolites in CRP gradually decreases with delayed harvest (Wang and Liu, 2014). CRP products harvested in September exhibited the highest volatile oil content, reaching 8.94% (Pan, 2011). Currently, relatively few studies have investigated the composition of CRP across different harvest periods. Future research should focus on this area to develop a more comprehensive understanding of CRP under the influence of various factors.

## 3 Microbial activity in CRP

During the storage of CRP, environmental microorganisms (including specific bacteria and fungi) play a crucial role in the transformation of bioactive metabolites. They act on specific metabolites through fermentation, with their activity and effects influenced by various environmental factors (Wu et al., 2013). The microbial community is a key determinant of quality differences in CRP (He et al., 2021).

### 3.1 Dominant bacteria

The microbial communities in CRP are diverse, with various microorganisms such as *Bacillus*, *Lactococcus*, *Pseudomonas*, *Aspergillus*, and *Penicillium* participating in the structural transformation of active metabolites (Wang Q. et al., 2023). The bacterial population in CRP mainly consists of *Bacillus*, *Lactococcus*, *Pseudomonas*, *Pontibacter*, *Carnobacterium*, and *Enterobacter*. Among these, *Bacillus* and *Lactococcus* are considered dominant genera and play significant roles in the production of D-limonene, hesperidin, and tangeretin (He et al., 2019). However, after different storage periods, the species composition and relative abundance of bacteria remain stable. Research on their role in relation to active metabolites is limited, as most studies have primarily focused on fungi (Wang Q. et al., 2023; Liu, 2019).

**FIGURE 4:**
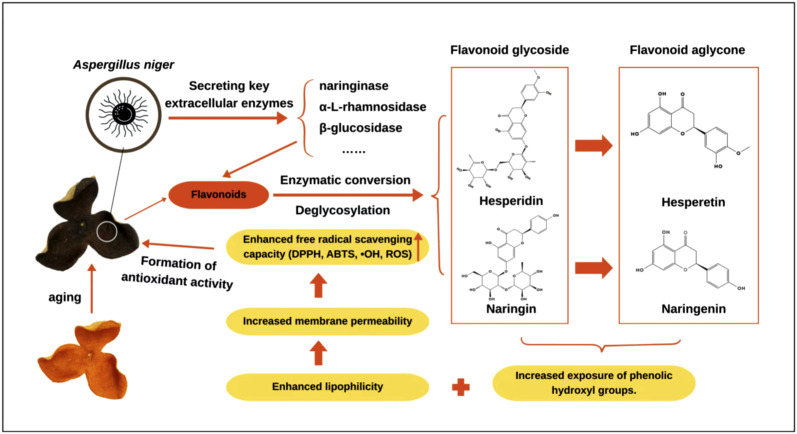
Mechanism of flavonoid transformation in CRP promoted by *Aspergillus niger*.

CRP harbors abundant fungal groups, including yeast, *Aspergillus*, *Penicillium*, *Alternaria*, *Wallemia*, *Cystofilobasidium*, *Zasmidium*, *Cladosporium*, *Hanseniaspora*, *Fusarium*, *Kurtzmaniella*, *Candida*, *Passalora*, *Ceramothyrium*, and *Afrocatena* (Liu, 2019). The fungal community composition changes with storage time. During the first year of storage, *Penicillium* is the dominant fungus in CRP (Yang et al., 2021). Over time, *Aspergillus niger* gradually becomes the predominant fungus, a finding confirmed through isolation and identification (Wang et al., 2018). *Aspergillus niger* is mainly distributed on the surface of CRP, typically appearing as dark brown or dark green colonies, thereby affecting the appearance and quality of CRP (Figure 4). This fungus promotes the transformation of flavonoids in CRP, converting naringin to hesperidin and further to hesperitin (Karim et al., 2022; Liu, 2019). The transformation mechanism of flavonoids by *Aspergillus niger* primarily involves enzymatic conversion. *Aspergillus niger* can secrete various extracellular enzymes, such as naringinase, α-L-rhamnosidase, and β-glucosidase. These enzymes hydrolyze the terminal glycosyl groups of flavonoid glycosides, converting them into more absorbable aglycones (Cao et al., 2015). A study by Ni et al. (2012) reached a similar conclusion, finding that α-L-rhamnosidase secreted by *Aspergillus niger* could hydrolyze hesperidin to hesperitin. Zou et al. (Zou et al., 2020) further confirmed that *Aspergillus niger* possessing both α-L-rhamnosidase and β-glucosidase activities could efficiently hydrolyze flavonoid glycosides including hesperidin, rutin, and naringin through whole-cell catalysis. This enzymatic transformation leads to the accumulation of polyhydroxyflavones in CRP and is considered one of the scientific connotations underlying the principle that “the longer CRP is preserved, the better the quality is.” To verify the promoting role of *Aspergillus niger* in this transformation, Liu et al. (2023) inoculated tangerine peels with *Aspergillus niger* and fermented them for 120 h. The results showed that with prolonged fermentation time, hesperidin content gradually decreased while hesperitin accumulated, total flavonoid content in the peels increased, and their antioxidant activity was significantly enhanced. Wang et al. (2021) compared original CRP (CK) with CRP fermented by *Aspergillus niger* (CP) and found that the free radical scavenging activity of the latter was significantly higher. Research by Wang et al. also confirmed that the antioxidant activity of CRP is closely related to the content of *Aspergillus niger* (Wang et al., 2018). The enhancement of antioxidant activity is mainly attributed to changes in flavonoid structure during fermentation. Specifically, the transformed aglycone flavonoids and polyhydroxyflavones possess more phenolic hydroxyl groups, which are key structures directly involved in free radical capture and electron transfer, thereby enhancing the overall antioxidant activity (Wang et al., 2018). The abundance of various fungal genera changes with storage time, with dominant genera succeeding each other at different stages; however, *Aspergillus niger* maintains its dominant position throughout the entire storage period (Yang et al., 2021).

### 3.2 Quality control of CRP

Strict quality control is essential for ensuring the efficacy of CRP. During storage, temperature and humidity affect tangerine peel, facilitating the growth and reproduction of *Aspergillus*, *Penicillium*, and other molds, which can degrade the quality and efficacy of CRP (Wang Q. et al., 2023). These molds may produce toxic metabolites, including aflatoxins, posing serious risks to human health (Zhang et al., 2024). According to the *Pharmacopoeia of the People’s Republic of China* (2020 edition), the limit for aflatoxin B_1_ in CRP is 5 μg/kg, and the combined total of aflatoxins G_2_, G_1_, and B_2_ must not exceed 10 μg/kg (Zhang et al., 2024). Furthermore, prolonged storage allows microorganisms to transform the chemical composition of CRP through their metabolic activities. Therefore, strict quality control of CRP in accordance with regulatory standards is essential to preserve its medicinal and food homology value.

## 4 CRP product development

**FIGURE 5:**
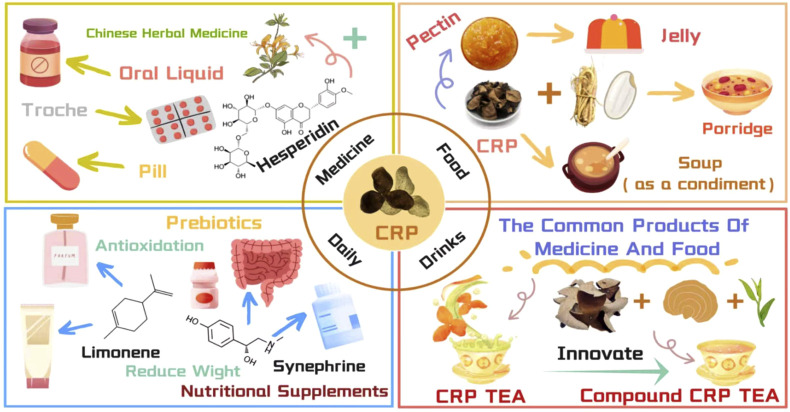
Application expansion and product development of CRP.

As an ingredient in traditional Chinese medicinal botanical drugs with a long medicinal history and unique flavor, CRP has demonstrated great application potential in traditional Chinese medicine, food, beverages, cosmetics, and other industries (Figure 5) (Peng et al., 2019; Waris et al., 2022). With growing attention to health and natural products, the development and utilization of CRP products have gradually become a research hotspot, aimed at fully exploring its value and meeting the diversified needs of the market.

### 4.1 Pharmaceutical field

Currently, CRP-containing products are commonly prescribed in the form of oral fluids, granules, boluses, and capsules (Yu et al., 2018). In these formulations, the active ingredients from CRP, such as flavonoids, volatile oils, and alkaloids, are extracted and combined with other Chinese medicinal materials or nutrients to produce drugs with specific therapeutic effects.

In the treatment of functional dyspepsia, Li et al. (2011) demonstrated through a randomized controlled trial that 20-year-aged Xinhui CRP significantly alleviated symptoms such as abdominal distension and loss of appetite. The clinical total effective rate reached 90.32% in the CRP monotherapy group and 94.12% in the combination therapy group, providing clinical evidence for its long-term application in digestive dysfunction. Furthermore, Hu et al. (2025) revealed through animal experiments that CRP intervention for 14 days improved body weight, gastric emptying rate, intestinal propulsion rate, and gastric histopathological structure in a rat model of functional dyspepsia. The underlying mechanism was closely associated with the regulation of the TLR4/MyD88 and MAPK inflammatory signaling pathways, as well as the remodeling of intestinal microbiota structure.

In the field of liver injury and metabolic liver diseases, the primary active flavonoids in CRP, particularly hesperidin, play a pivotal role in anti-inflammatory and metabolic regulation. Omar et al. (2016) demonstrated in a cisplatin-induced liver injury rat model that hesperidin (100, 200 mg/kg) attenuated inflammatory responses by inhibiting the NF-κB signaling pathway, reducing serum ALT and AST activities as well as hepatic MDA levels. A randomized controlled trial by Yari et al. (2021) indicated that hesperidin combined with flaxseed improved glucose and lipid metabolism, reduced certain inflammatory factors and hepatic fat content in patients with non-alcoholic fatty liver disease, and exerted synergistic effects on insulin resistance. Furthermore, Zou et al. (2024) revealed that CRP-derived exosome-like nanovesicles alleviated type 2 diabetes-associated hepatic lipotoxicity by activating genes related to fatty acid β-oxidation and glycolysis, thereby ameliorating insulin resistance and reducing hepatic lipid accumulation. These nanovesicles also contributed to restoring the intestinal barrier and modulating bile acid metabolism. Additionally, CRP-based metabolite preparations have been shown not only to improve non-alcoholic fatty liver disease but also to alleviate secondary constipation associated with type 2 diabetes. This effect may be attributed to the volatile oils and amino acids present in CRP, which stimulate gastric secretion and promote bile secretion and excretion, thereby reducing the burden on the hepatobiliary system (Shen et al., 2023).

In inflammatory gastrointestinal diseases, CRP has also demonstrated promising immunomodulatory potential. Cao et al. (2023) discovered in a mouse model of food-allergic eosinophilic esophagitis that CRP extract inhibited Th2-type immune responses (reducing IL-4 and IL-5) and alleviated tissue fibrosis by downregulating the MAPK/TGF-β signaling pathway, suggesting its therapeutic potential in food-induced inflammatory esophageal disorders. Research by Lee et al. (2024) further indicated that CRP-containing metabolite preparations suppressed the release of pro-inflammatory cytokines (TNF-α, IL-1β, IL-6, IFN-γ) while promoting the expression of anti-inflammatory factors (IκB-α, IL-10, IL-4), demonstrating clear immunomodulatory effects. In clinical applications for chronic atrophic gastritis, a CRP-containing metabolite ointment showed significant efficacy, with a total clinical symptom remission rate of 94.0% and a pathological improvement rate of 80.0% (Yu et al., 2018).

In conclusion, as a traditional Chinese medicine, CRP and its active metabolites, particularly hesperidin, exhibit well-defined pharmacological effects in regulating digestive function, protecting against liver injury, improving metabolic liver diseases, and inhibiting gastrointestinal inflammation. It demonstrates clinical potential for multi-target regulation of inflammatory pathways and intervention in metabolism and immune responses (Choi et al., 2022). However, current research primarily focuses on flavonoids, and the medicinal value of other chemical constituents in CRP warrants further investigation.

### 4.2 Food field

Due to its unique flavor and chemical composition, CRP has been widely used in food modification, the development of novel snacks and tea beverages, and various other application areas (Shi et al., 2024). In food industry applications, CRP is rich in pectin (Tamaki et al., 2008), which not only imparts desirable texture to foods but also enables it to function as a gelling agent, thickener, and stabilizer (Shi et al., 2024). Based on these properties, a research team developed a functional kiwifruit jelly enriched with CRP. This product is abundant in phenolic metabolites, low in calories, and exhibits antioxidant and anti-inflammatory effects, effectively inhibiting fat accumulation (Peng et al., 2022). Furthermore, a study by Teixeira et al. (2020) demonstrated that incorporating CRP into marmalade significantly increased the vitamin C content from 124.83 mg/100 g to 150.12 mg/100 g, providing a new approach for developing functionally fortified foods. In the realm of traditional medicinal cuisine, CRP also plays a significant role. “CRP porridge,” prepared by boiling milk grassroots, stem rice, and CRP, is traditionally believed to possess the effects of replenishing qi and beautifying the complexion. The renowned traditional Cantonese dish “braised pigeon with CRP” is thought to promote blood circulation, making it a popular medicinal meal suitable for patients suffering from arrhythmias and palpitations (Yu et al., 2018). Additionally, the sweet and mellow characteristics of CRP render it a widely used culinary adjunct, establishing it as an indispensable flavoring agent in tonics and soups (Yu et al., 2018). It is worth noting that the application scenarios of CRP are continuously expanding into emerging fields. An experiment by Wang et al. innovatively incorporated CRP as a functional ingredient into canine snacks, revealing that it significantly enhances serum antioxidant enzyme activity and improves intestinal health in dogs (Wang et al., 2025). This provides a reference for exploring the potential of CRP in the functional pet food sector.

Similarly, CRP possesses a fruity citrus aroma and can serve as a flavor enhancer in beverages to improve both the taste and nutritional properties of products (Guichard et al., 2019). Furthermore, CRP has been confirmed to be rich in flavonoids, exhibiting significant antioxidant activity and the ability to ameliorate digestive and metabolic disorders, helping to scavenge free radicals and protect gastrointestinal health (Shi et al., 2024). Based on its unique flavor profile and health benefits, incorporating CRP extracts into fruit juices and carbonated beverages to develop products with distinctive flavors or appetite-stimulating effects holds significant potential for strengthening the beverage market. CRP tea is a common medicinal and edible product with the traditional efficacy of moistening the lungs and benefiting the stomach. Its production process involves blending dried CRP with Pu’er tea, placing the mixture in a ventilated and cool environment for natural air-drying, and subsequently steeping it in boiling water (Zheng et al., 2020b). This processing technique integrates the essence of both Pu’er tea and CRP, allowing the tea leaves to fully absorb the rich fruity aroma of CRP, which perfectly complements the mellow flavor of Pu’er tea. Concurrently, its properties of regulating qi and strengthening the spleen can help improve poor appetite and abdominal distension, thereby promoting digestion and absorption. CRP tea has consistently attracted interest among researchers in the field of medicine and food homology, and in recent years, the development of compound formulations has further expanded its applications. For example, Compound CRP Tea (CCT), formulated by combining CRP, *Ganoderma lucidum*, and Pu’er tea in a 1:1:1 ratio, has been shown to effectively reduce obesity rates, alleviate hepatic steatosis, and ameliorate dyslipidemia compared to traditional tangerine peel tea (Wang J. et al., 2023). Beyond tea-based beverages, Jiangsu’s renowned “CRP wine,” whose history can be traced back to the Qing Dynasty, is rich in various amino acids and other nutrients, offering significant value in promoting qi and blood circulation and regulating meridians (Yu et al., 2018).

### 4.3 Other applications

CRP has numerous well-established applications in the pharmaceutical and food industries. However, with more in-depth research and development, its potential can be extended to a broader range of sectors (Waris et al., 2022). For instance, the volatile oils and flavonoids extracted from CRP exhibit significant application value in commonly used chemical products such as perfumes and skincare items (He and Xiao, 2016). These extracts are not only rich in the active ingredients characteristic of essential oils but also impart a unique and pleasant aroma (Giatropoulos et al., 2012). Furthermore, their antioxidant properties enable them to form a protective film on the skin’s surface, mitigating damage from external oxidative factors and helping to maintain the skin’s moisture and oil balance. Synephrine, another key metabolite, is frequently incorporated into nutritional supplements for appetite control and weight management. When used under medical guidance, it may promote metabolism (Ruiz-Moreno et al., 2021). This finding offers valuable insights for the future application of CRP in the healthcare product sector. Notably, the effect of combining CRP with probiotics resembles that of prebiotics. In such applications, CRP not only aids in regulating gastrointestinal function but also works synergistically with probiotics to maintain intestinal flora balance, promote the colonization and proliferation of beneficial bacteria, and subsequently enhance intestinal immunity (Wang Q. et al., 2023). Currently, research on the application of CRP in other industries remains limited; therefore, these potential innovations provide promising directions for future investigation.

## 5 Conclusions and future perspectives

CRP not only carries a profound cultural heritage but also constitutes an important component of modern healthcare, being widely utilized in various medicine and food products as well as functional foods. In particular, CRP produced in Xinhui, Guangdong Province, is renowned both domestically and internationally for its exceptional flavor and quality. This review synthesizes previous research findings and innovatively systematically compares the quality differences of CRP across different storage times, aging times, production areas, and harvest periods. It reveals how these factors regulate the dynamic changes in the chemical composition and bioactivity of CRP, thereby providing a new perspective for deepening the scientific understanding of CRP’s medicine-food homology characteristics. The review also discusses the variation trends in the content of major active metabolites, such as hesperidin, D-limonene, and synephrine, in response to various influencing factors, confirming the decisive role of these factors in determining the ultimate efficacy of CRP. Furthermore, this review systematically elaborates on the critical role of microbial transformation mechanisms in the quality formation of CRP—specifically, *Aspergillus niger*, as a dominant strain, can effectively promote the biotransformation of flavonoid metabolites, thereby enhancing the antioxidant, anticancer, and anti-inflammatory effects of CRP to a certain extent. This finding provides a scientific basis for interpreting the traditional understanding that “CRP improves with age” and opens a new avenue for optimizing the functional properties of CRP through directed microbial regulation.

Based on existing research, this review proposes the following priorities for future investigation: (1) In-depth analysis of microbial transformation mechanisms: Elucidate the specific metabolic pathways through which *Aspergillus niger* and other dominant strains mediate the biotransformation of active metabolites during CRP aging, and explore the potential for directed regulation of microbial communities to enhance CRP efficacy. (2) Synergistic effects of storage times and aging processes: Systematically characterize the dynamic changes of additional chemical constituents in CRP over aging times, and investigate the interplay between general storage duration and the aging process and their differential impacts on CRP quality. (3) Exploitation of emerging CRP varieties: Identify the differences in active metabolites and quality among various CRP cultivars from more production regions, systematically evaluate their functional properties, and promote the discovery and utilization of CRP varieties with development potential. (4) Establishment of standardization and safety assessment systems: Develop content determination standards based on characteristic active metabolites and safety indicators (e.g., aflatoxins) for CRP from different aging years and geographical origins, and establish comprehensive quality control protocols to ensure product quality and stability. (5) Exploration of emerging application fields: Investigate the potential applications of CRP in high-value-added sectors such as cosmetics and healthcare products, and develop functional products based on targeted delivery of active metabolites to expand the industrialization pathways of CRP.

**FIGURE 6:**
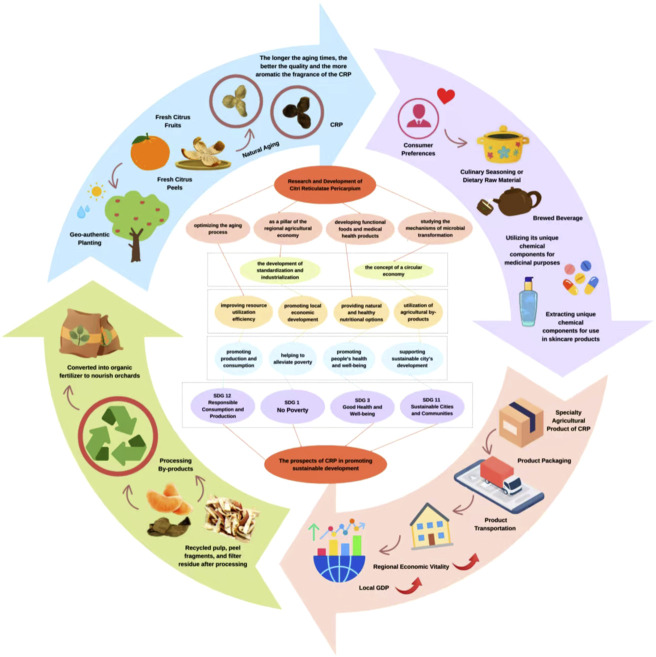
The roadmap of CRP’s role in the SDGs.

Notably, the research and development of CRP align closely with the United Nations Sustainable Development Goals (SDGs) (Figure 6). First, optimizing aging processes to enhance the resource utilization efficiency of CRP contributes to promoting responsible consumption and production (SDG 12). Second, the CRP cultivation industry serves as a vital agricultural economic pillar in regions such as Xinhui, Guangdong Province; its standardization and industrial development can stimulate local economic growth and aid in poverty eradication (SDG 1). Third, functional foods and healthcare products developed based on the active metabolites of CRP offer consumers natural and healthy nutritional options, thereby contributing to the promotion of good health and wellbeing (SDG 3). Finally, the research and application of microbial transformation mechanisms align with the principles of the circular economy, enabling the high-value utilization of agricultural by-products through biotechnological approaches and supporting sustainable cities and communities (SDG 11). Therefore, in-depth investigation of CRP not only holds significant scientific value but also encompasses broad prospects for advancing sustainable development.

In summary, future in-depth research on the variation patterns of CRP’s chemical components and their underlying bioactive mechanisms under different influencing factors will provide a solid scientific foundation for the clinical translation of CRP and the standardized development of related functional products, while also opening new avenues for the sustainable utilization of traditional Chinese medicine resources.
